# Identification of Spinal Cord MicroRNA and Gene Signatures in a Model of Chronic Stress-Induced Visceral Hyperalgesia in Rat

**DOI:** 10.1371/journal.pone.0130938

**Published:** 2015-07-29

**Authors:** Sylvie Bradesi, Iordanes Karagiannides, Kyriaki Bakirtzi, Swapna Mahurkar Joshi, Georgios Koukos, Dimitrios Iliopoulos, Charalabos Pothoulakis, Emeran A. Mayer

**Affiliations:** 1 Oppenheimer Family Center for Neurobiology of Stress, Division of Digestive Diseases, David Geffen School of Medicine at UCLA, Los Angeles, California, United States of America; 2 Inflammatory Bowel Disease Center, and Neuroendocrine Assay Core, Division of Digestive Diseases, David Geffen School of Medicine at UCLA, Los Angeles, California, United States of America; 3 Center for Systems Biomedicine, Division of Digestive Diseases, David Geffen School of Medicine at UCLA, Los Angeles, California, United States of America; 4 CURE Center, Veterans Administration, Greater Los Angeles, California, United States of America; Central South University, CHINA

## Abstract

**Introduction:**

Animal studies have shown that stress could induce epigenetic and transcriptomic alterations essential in determining the balance between adaptive or maladaptive responses to stress. We tested the hypothesis that chronic stress in rats deregulates coding and non-coding gene expression in the spinal cord, which may underline neuroinflammation and nociceptive changes previously observed in this model.

**Methods:**

Male Wistar rats were exposed to daily stress or handled, for 10 days. At day 11, lumbar spinal segments were collected and processed for mRNA/miRNA isolation followed by expression profiling using Agilent SurePrint Rat Exon and Rat miRNA Microarray platforms. Differentially expressed gene lists were generated using the dChip program. Microarrays were analyzed using the Ingenuity Pathways Analysis (IPA) tool from Ingenuity Systems. Multiple methods were used for the analysis of miRNA-mRNA functional modules. Quantitative real time RT-PCR for Interleukin 6 signal transducer (gp130), the Signal Transducer And Activator Of Transcription 3 (STAT3), glial fibrillary acidic protein and mir-17-5p were performed to confirm levels of expression.

**Results:**

Gene network analysis revealed that stress deregulated different inflammatory (IL-6, JAK/STAT, TNF) and metabolic (PI3K/AKT) signaling pathways. MicroRNA array analysis revealed a signature of 39 deregulated microRNAs in stressed rats. MicroRNA-gene network analysis showed that microRNAs are regulators of two gene networks relevant to inflammatory processes. Specifically, our analysis of miRNA-mRNA functional modules identified miR-17-5p as an important regulator in our model. We verified miR-17-5p increased expression in stress using qPCR and in situ hybridization. In addition, we observed changes in the expression of gp130 and STAT3 (involved in intracellular signaling cascades in response to gp130 activation), both predicted targets for miR-17-5p. A modulatory role of spinal mir17-5p in the modulation of visceral sensitivity was confirmed in vivo.

**Conclusion:**

Using an integrative high throughput approach, our findings suggest a link between miR-17-5p increased expression and gp130/STAT3 activation providing new insight into the possible mechanisms mediating the effect of chronic stress on neuroinflammation in the spinal cord.

## Introduction

Sustained engagement of the stress system can lead to maladaptive responses, including the development and maintenance of chronic pain [1]. The responsiveness and recovery of the stress system is influenced by individual differences in genetic background supported by evidence of gene polymorphisms associated with vulnerability to stressors [2]. Epigenetic modulation, such as biochemical modifications of genomic DNA via methylation, histone modification, chromatin remodeling or small non-coding RNAs (microRNA, miRNA) [3] also constitutes another component of regulation of stress responsiveness [4]. Recent studies suggested that stress may affect the transcription, processing and turnover of microRNAs as well as the activities of microRNA-protein complexes, which in turn can alter the expression of mRNA targets [5]. The relative position of a specific microRNA within a gene circuit, and its modulation by environmental factors, may affect feedback loops shaping a new gene expression pattern defining cellular fate.

In the recent years, animal studies have pointed to an effect of stress on the neuroinflammatory response in the central nervous system (CNS) supporting stress-induced modulation of CNS microglia immunophenotype [6] and increasing evidence indicates an important role of spinal microglia and astrocytes in the modulation of nociceptive sensitivity in animal models of chronic pain [7–9]. Only few studies have investigated the role of spinal glia activation and neuroinflammation in visceral pain [10–12]. We have previously demonstrated that rats exposed to chronic psychological stress (1 hour daily exposure to water avoidance stress) show increased anxiety behaviors and increased visceromotor response to colorectal distension as an indication of visceral hyperalgesia. Our studies confirmed the role of spinal glia in this effect and observed a modulatory influence of stress on the expression of various spinal molecules involved in nociceptive signaling pathways. Notably, a decreased expression of spinal glial fibrillary acidic protein (GFAP) was observed after stress, associated with changes in the expression of several molecules related to glutamatergic signaling (excitatory amino acid transporter EAAT2 (GLT1), EAAT2 (GLAST), Glutamine synthetase [13] or several pro-inflammatory cytokines including Interleukin-1ß (IL-1ß), IL-6 and Tumor necrosis factor alpha (TNF-alpha).

The current study aimed to test the general hypothesis that chronic stress-induced changes in spinal glia, which underlie visceral hyperalgesia, are associated with changes in miRNA and protein encoding gene expression in a network or several connected sub-networks related to neuroinflammation. We demonstrate that chronic water avoidance stress in rats induces changes in networks of genes and miRNA in the spinal cord. We confirmed stress-induced changes in the expression of several mRNA and miRNAs and established a possible network linking mRNA and miRNA involved in the effect of stress on pain signaling.

## Materials and Methods

### Animals

Adult male Wistar rats (250–275 g; Harlan, Inc.) were maintained on a normal light-dark cycle, and provided with food and water ad libitum. All protocols were approved by the Institutional Animal Care and Use Committee at the VA Greater Los Angeles Healthcare System. All animal experiments were carried out in accordance with the National Institute of Health Guide for the Care and Use of Laboratory Animals (NIH Publications No. 80-23, revised 1978).

### Chronic water avoidance stress

The model of chronic WA stress consists in placing each animal on a small platform (8 × 8 × 10 cm) affixed to the center of a Plexiglas cage (25 × 25 × 45 cm) filled with tepid water (25 degree Celsius) to within 1 cm of the top of the platform for 1 h per session. Animals are not directly exposed to the water and when placed on the platform, avoid the aversive stimulus (water) by remaining on the platform. The rats were placed on the platform and in case they fell, they were immediately removed from the water, dried, and placed again on the platform. The rats learned quickly to avoid the water and never fell more than once or twice and if so, only during the first exposure to the stressor. The model of chronic WA stress (10 days) is a validated model of chronic psychological stress-induced visceral hyperalgesia and has been described in detail previously [14,15].

### Spinal cord dissection, RNA extraction and mRNA and mirRNA arrays

Rats were anesthetized with Isoflurane (Baxter, IL) and quickly decapitated. The spinal cord was collected by hydroextrusion with iced saline, the meninges were removed and lumbar L6S1 spinal segments were dissected immediately. mRNA and miRNA were isolated from spinal tissues using the RNeasy mini kit (QIAGEN). Gene expression and mirRNA profiling were performed at the UCLA Clinical Microarray Core using the Agilent 028279 SurePrint G3 Rat GE 8x60K and Agilent 019159 Rat miRNA Microarray 8x15K platforms, respectively.

### In mir17-5p situ hybridization with co-immunofluorecent staining for GFAP

Rats exposed to stress or control rats were deeply anesthetized and perfused intracardially with 0.9% saline followed by a solution of 4% paraformaldehyde (PFA). The lumbar spinal cord (L6S1) was dissected, post fixed in 4% PFA, cryoprotected in 20% sucrose and embedded in Tissue-Tek O.C.T. on dry ice. 14μM cryostat sections were collected and stored at -80 degree Celsius. Tissue sections were processed as recommended in the Exiqon miRCURY LNA microRNA ISH optimization kit. For hybridization, 0.15nmol/ml of hsa-mir17-5p miRCURY LNA detection probe (88084-15) or positive control mir-124 miRCURY LNA detection probe and negative scramble control probe (90004) were incubated in hybridization buffer at 55 degree Celsius for one hour in an hybridization oven. Slides were then washed in SSC buffer, blocked in blocking buffer before incubation overnight with the alkaline phosphatase-conjugated anti-digoxigenin antibody (1:800, Roche Diagnostics GmbH) at 4 degree Celsius. Slides were then washed and blocked in NGS 10% before incubation overnight with a mouse primary antibody for GFAP (1:1000, Millipore, MAB360). Sections were then rinsed in PBS and incubated for 4 hours at room temperature with Alexa Fluor 488-conjugated secondary antibodies (1:800, Invitrogen A11029). The immunological detection of anti-digoxigenin antibody was performed using either the NBT-BCP detection system or the HNPP Fluorescent detection set (Roche Diagnostics, GmbH) according to manufacturer’s instructions. Sections were rinsed and mounted in Vectashield mounting medium + DAPI (Vector laboratories, H-1200) and observed using an Axio Observer Z1 microscope equipped with the Apotome system (Zeiss, Germany).

### mRNA and miRNA microarrays analysis

#### mRNA microarray analysis

We performed a whole genome transcription profiling of spinal cord samples from control (n = 4) and stressed rats (n = 4). Differentially expressed genes lists were generated using the dChip software program. The resulting expression patterns were analyzed using the Ingenuity Pathway Analysis (IPA) tool from Ingenuity Systems. Given a list of genes, IPA performs a statistical test for enrichment of these genes in its hand-curated canonical pathway database. Each individual IPA signaling pathway includes genes that have been described to interact in the published scientific literature. These tools allow us to determine the top networks, biological functions and canonical signaling pathways represented among the genes that are significantly enriched in tissue from stressed animals.

#### miRNA microarrays analysis

MiRNA-associated gene repression has been shown to be involved in the regulation of almost every single intracellular pathway identified, thus playing a role in the pathogenesis of many disease states. MiRNA microarray analysis was performed using the dChip software program to generate a list of differentially expressed miRNA in control and stressed animals.

### Quantitative real time PCR for genes and miRNA

RNA was isolated from spinal cord tissue using the Trizol method. 1 μg of RNA isolated was reverse-transcribed into cDNA and incubated with dual fluorogenic probes (Applied Biosystems, Foster City, CA). GAPDH was used as endogenous controls and was also detected using dual labeled fluorogenic probe (5’-FAM/3’-MGB probe, Applied Biosystems, Foster City, CA). The miRCURY LNA universal RT microRNA PCR kit using RNU1A1 as endogenous control was used for mir real time RT-PCR. Target mRNA (IL6ST, IkBib and Hmbgb1, STAT3) and mir (mir17-5p) (Applied Biosystems) levels were quantified using a fluorogenic 5'-nuclease PCR assay using a 7500 Fast Real-Time PCR sequence detection system (Applied Biosystems, Foster City, CA).

### Analysis of predicted miRNA targets

Multiple methods were used for the analysis of miRNA-mRNA functional modules. First, we predicted targets of the 39 differential miRNAs using miRWALK [16]. Then, we performed an analysis of the miRNA predicted targets in our 70 genes list using the Ingenuity miRNA target filter. In addition, we used DNA Intelligent Analysis (DIANA) tools to further analyze targets for the mir17-5p. TarBase 6.0 is the largest available manually curated database of experimentally supported miRtargets. The database includes targets derived from specific and high throughput experiments such as microarray and proteomics as well as PCR and western blot. The database is seamlessly interconnected with other DIANA-lab tools, such as DIANA-microT, enabling it to extend each validated interaction with in silico predicted information. Currently, the TarBase 6.0 dataset is freely available for download. DIANA-TarBase 6.0 can be accessed from the following address: /DianaTools/index.php?r=tarbase/index. Next, we performed an analysis of expression data for microRNA function using the DIANA0mirExTra tool. DIANA-mirExTra is an algorithm that can identify microRNA effects to the expression levels of protein-coding transcripts, based on the frequency of six nucleotide long motifs (hexamers) in the 3'UTR sequences of genes. Direct links to further functional analysis of produced results based on DIANA-mirPath are provided for all results. DIANA-mirExTra can be accessed from the following address: http://diana.cslab.ece.ntua.gr/hexamers

### Testing the effect of the mir17-5p inhibitor on stress-induced visceral hyperalgesia

#### Surgical implantation of chronic intrathecal catheter and osmotic mini-pump

Rats were anesthetized with Nembutal (Abbot Laboratories, North Chicago, IL, USA; 50mg/kg, I.P.) and surgically equipped with a chronic intrathecal (I.T.) catheter (8.5 cm polyethylene tubing-5, OD 0.14', ID 0.006', Spectranetics, Colorado Springs, CO, USA), connected to a 4 cm PE10 (polyethylene tubing-10), inserted through the atlanto-occipital membrane of the cisterna magna. Following surgery, rats received S.C. injection of buprenorphine (0.03mg/kg) and were monitored for 3 days and allowed to recuperate for at least 5 days before CRD testing. Wounds were tested for tenderness to ensure complete recovery from surgery prior to testing. Rats exhibiting any sign of neurological or motor impairment, as evidenced by paralysis, abnormal gait, weight loss, or negligent grooming, were excluded from the study and euthanized. For chronic I.T. treatment, the tip of the catheter was connected to an osmotic minipump (Alzet, model 2002, Cupertino, CA) positioned under the skin between the scapulas. Catheters were primed with vehicle/drugs before implantation. After completion of testing, the catheter position was verified in each animal by postmortem examination of the spinal cord.

#### Surgical implantation of electromyographic (EMG) electrodes and assessment of visceromotor response (VMR) to colorectal distension (CRD)

EMG electrodes (Teflon-coated stainless steel wire, Cooner Wire, CA) were stitched into the external oblique musculature, for electromyographic (EMG) recordings as previously described[13]. The visceral stimulus employed was distension of the descending colon and rectum using a well established and validated method[13]. Briefly, under light Isoflurane® anesthesia, a flexible latex balloon (6 cm) was inserted intra-anally (after the distal part of the rectum was gently cleared by massage) such that its end was 1 cm proximal to the anus. Once recovered from anesthesia, animals equipped with the balloon were placed in a Plexiglas cylinder for 30 min before the CRD procedure was initiated. The CRD procedure consisted of two series of phasic CRDs to constant pressures of 10, 20, 40, and 60 mm Hg (20 s duration; 4-min inter-stimulus interval). The VMR to CRD was quantified by measuring EMG activity in the external oblique musculature. EMG activity was recorded 20 s before (baseline), 20 s during, and 20 s after termination of CRD. The EMG activity was rectified, and the increase in the area under the curve [17] of EMG amplitude during CRD over the baseline period before CRD was recorded as the response. In the following, we will use the term EMG referring to the VMR to CRD.

Two groups of stressed rats were used to test the effect of the miRCURY LNA mir17-5p inhibitor probe (Exiqon, Cat #199900, CTGTAAGCACTTTG) or the scramble negative control probe (ACGTCTATACGCCA) on visceral sensitivity in stressed rats. Treatments were delivered via the osmotic minipump and the I.T. catheter at a concentration of 2μg/day, 12 μL/day for the whole duration of the stress procedure and CRD testing.

### Statistical analyses

Initial processing of microarray and miRNA data was performed using dChip IPA [18]. Hierarchical clustering and heatmaps were generated using R package *heatmap*.*plus (* *http*:*//cran*.*us*.*r-project*.*org/* *)*. We performed bicluster analysis to investigate integrated mRNA and miRNA functional modules using miRMAP, as described by Bryan et al [19]. For the qRT-PCR results, statistical significance was tested using an unpaired t-test and significance was achieved when P<0.05. Visceral pain data were analyzed using a two way ANOVA followed by a Bonferroni post test. P values are indicated in the graphs.

## Results

### Identification of gene networks deregulated in stressed rats

**Fig 1:**
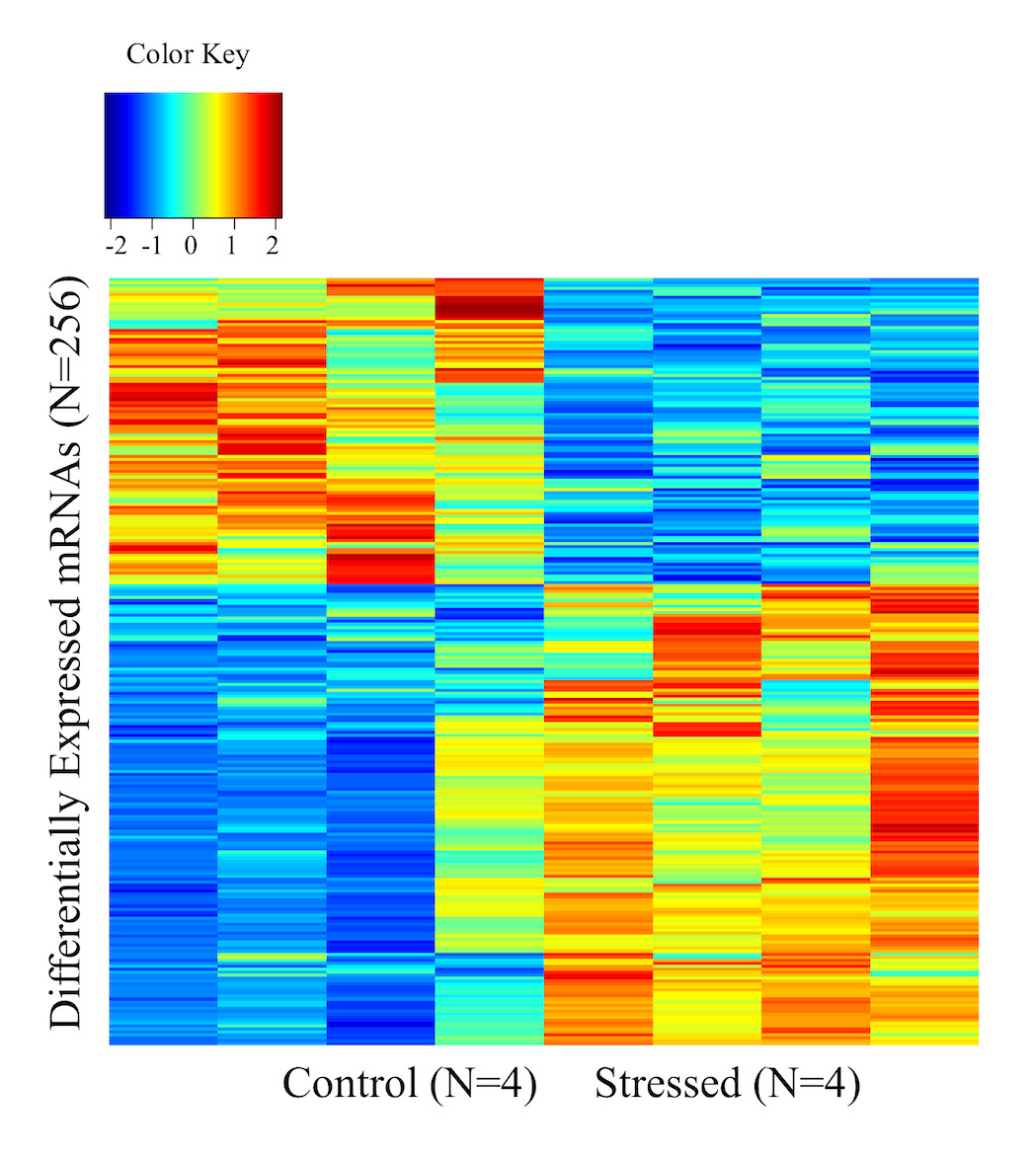
Heatmap showing unsupervised clustering of expression Z-scores of 256 unique genes from the list of 701 differentially expressed mRNAs.

The dChip analysis of our mRNA array generated a list of 701 differentially expressed genes between stressed and control animals, by using an FDR at <0.1. The mRNA expression (Z-scores) of the 701 differentially expressed genes (256 unique genes) between stressed and control animals was visualized using heatmap analysis as shown in Fig 1. Next, we performed gene network analysis by using the IPA software for the genes that were differentially expressed between the stressed and control animals. This analysis revealed 6 statistically significant (score>25) networks. Among these networks, the top network (score 41) included 41 molecules involved in Cellular Function and Maintenance, Inflammatory Response, Cell-To-Cell Signaling and Interaction and Inflammatory Response.

**Fig 2:**
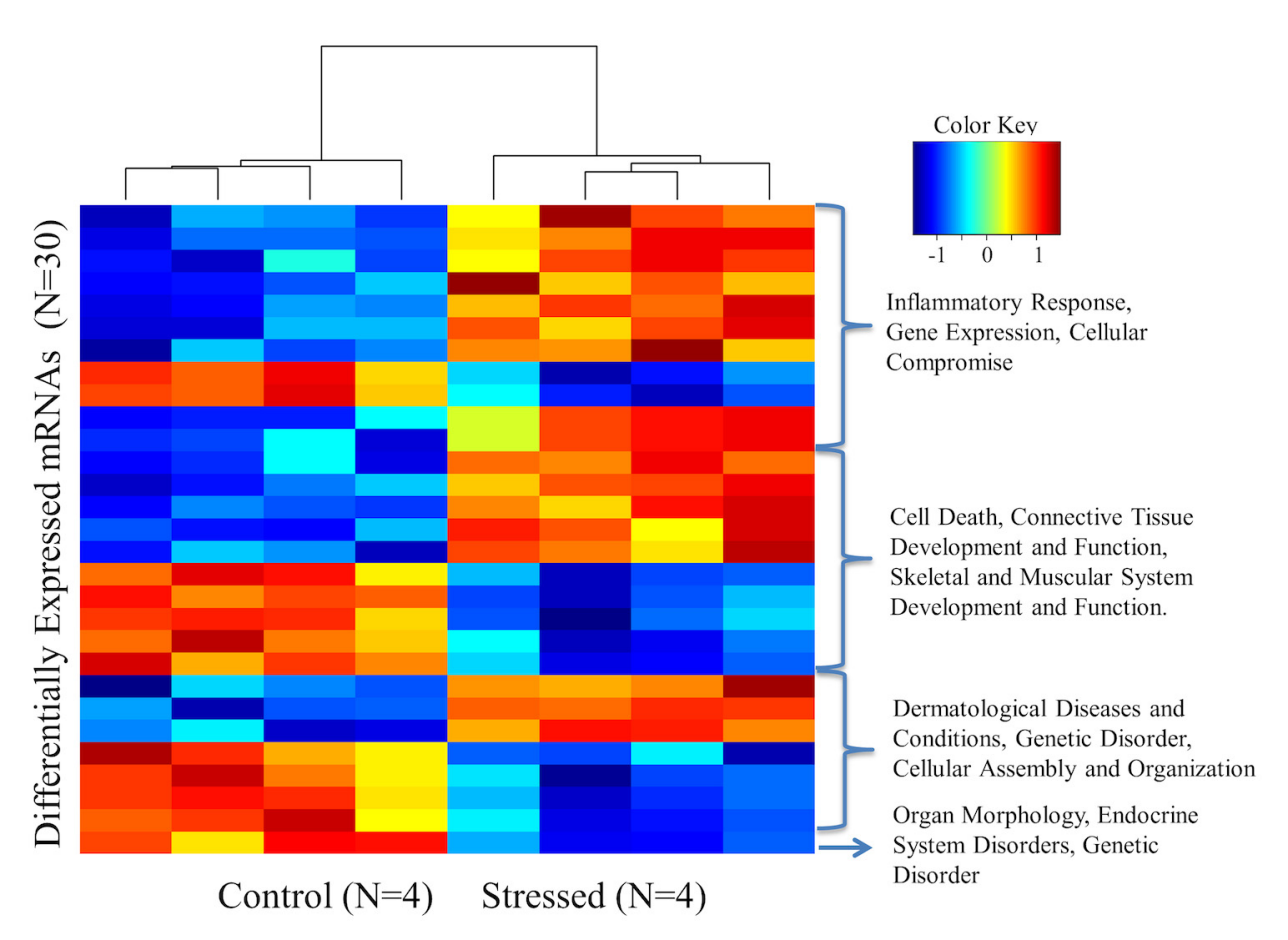
Heatmap of expression Z-scores of 30 unique genes from the 70 differentially regulated gene list.

In order to refine our analysis, we strengthened our selection criteria and used a more stringest FDR (FDR<0.05). These stringent criteria generated a list of 70 genes, which were clustered relative to their biological function (Fig 2). Gene network analysis of this new 70-gene signature identified 2 statistically significant networks with scores of 38 and 29, respectively. Interestingly, these networks included molecules involved in Cell Death and survival, Gene Expression, RNA damage and repair (Network 1) and Connective Tissue Disorders, Inflammatory Disease, Skeletal and Muscular Disorders (Network 2) as shown in Table 1.

**Table 1:** Networks of molecules identified from IPA analysis of the 70 genes list.

ID	Molecules in Network	Focus Molecules	Top Functions
1	AGMAT,BMP2,C6orf211,CCDC58,DNAJC21,ELAVL1,FAM49B,GLOD4,GPRC5C,HIC2,HNF4A,HSD17B12,HSDL2,KCNC3,KIAA1551,KNTC1,L2HGDH,LAMA5,MAPK1,MLEC,MRTO4,NBAS,PCNP,PITPNB,PTRH2,RSBN1,RSL24D1,SCFD2,TNN,UBC,VMA21,ZC3H15,ZFP37,ZNF528,ZWILCH	15	Cell Death and Survival, Gene Expression, RNA Damage and Repair
2	Akt,CCR2,CDK4/6,CDKN2B,CLCF1,CMTM8,DHX9,ERK,ERK1/2,GDF15,gelatinase,GMFB,GPRC5C,HAMP,Hmgb1,ID4,IFNL2,IKBKE,IL6ST,Interferon alpha,LGI1,MMP11,NEDD9,Neuropilin,NFkB (complex),OSMR,PDGF-AA,PI3K (complex),PLAC8,PTRH2,SEMA3F,Stat1/3,Tgf beta,Vegf,Ybx1-ps3	12	Connective Tissue Disorders, Inflammatory Disease, Skeletal and Muscular Disorders

In addition to the network analysis, we performed gene ontology (GO) analysis using the IPA software and found that amongst the top canonical pathways were the IL-6 signaling pathway, PI3K/AKT signaling pathway and the acute phase response signaling pathway, all highly relevant to neuro-inflammation. The list of molecules involved in the GO analysis is included in Table 2. IL6ST was found to belong to all 3 of these canonical pathways.

**Table 2:** Canonical pathways generated by the IPA analysis of the 70 genes list.

Ingenuity Canonical Pathways	-log(p-value)	p vallue	Ratio	Molecules
Small Cell Lung Cancer Signaling	2.36E00	0.0044	2.25E-02	IKBKE,CDKN2B
Mouse Embryonic Stem Cell Pluripotency	2.11E00	0.00774	2.02E-02	IL6ST,ID4
IL-6 Signaling	1.94E00	0.0114	1.61E-02	IL6ST,IKBKE
PI3K/AKT Signaling	1.9E00	0.0127	1.39E-02	GDF15,IKBKE
Acute Phase Response Signaling	1.64E00	0.0231	1.11E-02	IL6ST,IKBKE
Role of JAK family kinases in IL-6-type Cytokine Signaling	1.46E00	0.0344	3.7E-02	IL6ST
TNFR2 Signaling	1.41E00	0.0385	3.03E-02	IKBKE
4-1BB Signaling in T Lymphocytes	1.37E00	0.042	2.78E-02	IKBKE
Colorectal Cancer Metastasis Signaling	1.35E00	0.0447	7.63E-03	IL6ST,MMP11
TWEAK Signaling	1.34E00	0.052	2.63E-02	IKBKE
Oncostatin M Signaling	1.33E00	0.065	2.86E-02	IL6ST
IL-17A Signaling in Fibroblasts	1.32E00	0.079	2.5E-02	IKBKE
April Mediated Signaling	1.28E00	0.051	2.33E-02	IKBKE
4-1BB Signaling in T Lymphocytes	1.37E00	0.042	2.78E-02	IKBKE
Colorectal Cancer Metastasis Signaling	1.35E00	0.0447	7.63E-03	IL6ST,MMP11
TWEAK Signaling	1.34E00	0.052	2.63E-02	IKBKE
Oncostatin M Signaling	1.33E00	0.065	2.86E-02	IL6ST

### Identification of a microRNA signature in stressed rats

**Fig 3:**
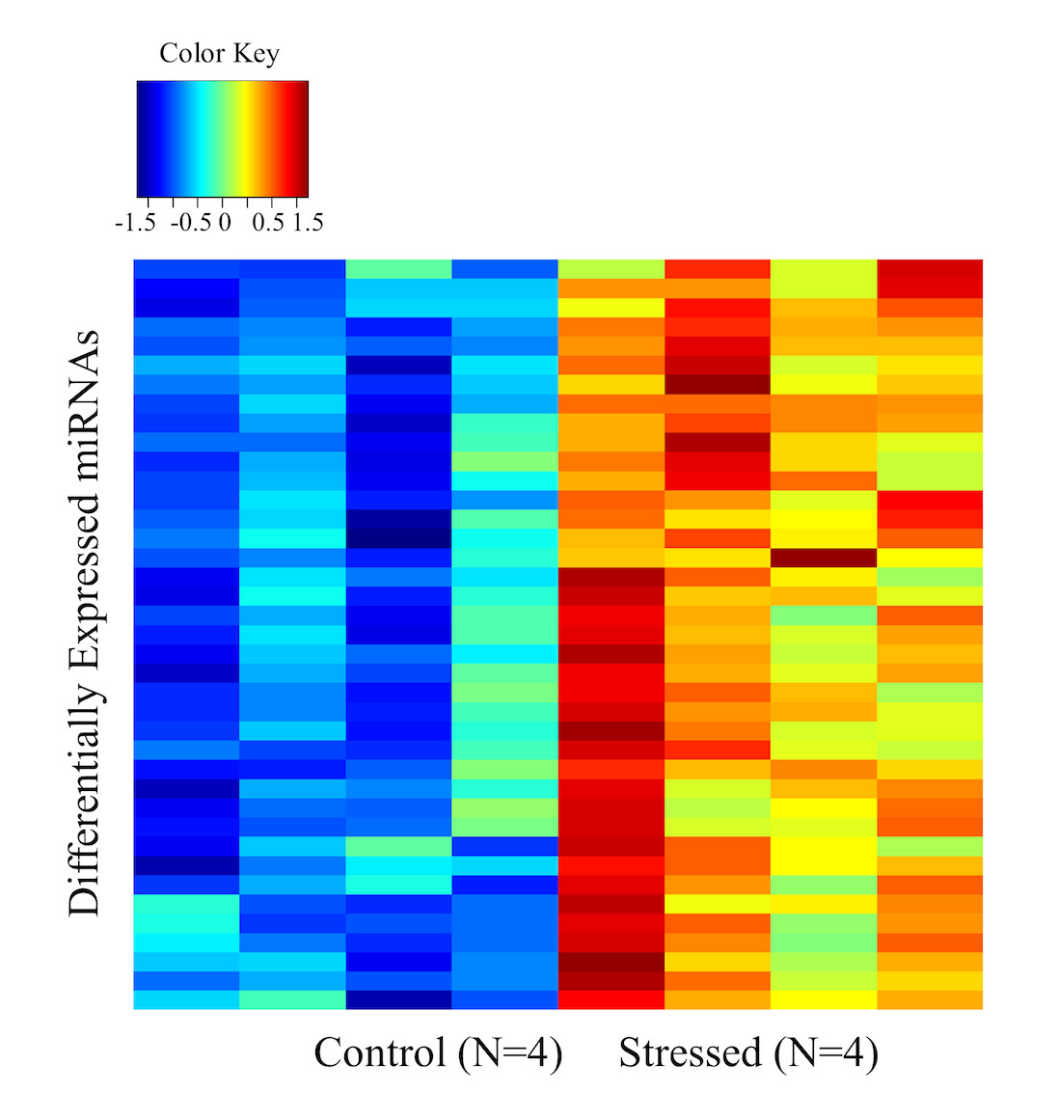
Unsupervised clustering of miRNA expression Z-scores.

MicroRNA array analysis revealed that 39 microRNAs are statistically significant (FDR<0.05) deregulated between control and stress groups. We performed unsupervised clustering of expression Z-scores of differentially expressed miRNAs, as shown in Fig 3 and the list of differentially expressed miRNA is shown in Table 3. We performed microRNA network analysis by using the IPA software for the 39 deregulated miRNAs and identified 2 networks (scores of 47 and 25) in which important functions such as inflammatory diseases were identified. The molecules and top functions associated with these networks are listed in Table 4.

**Table 3:** List of 39mirRNA differentially expressed in control and stressed samples.

mmu-miR-677	1.59063919
hsa-miR-516a-5p	1.846808511
rno-miR-219-5p	1.598183824
rno-miR-219-2-3p	1.552373074
mmu-miR-1939	1.312844612
mmu-let-7c-2*	1.699867198
rno-miR-30a	1.68225084
rno-miR-30e	1.63203197
rno-miR-19a	1.628297362
rno-miR-494	1.534276674
rno-miR-15b	1.529882353
mmu-miR-582-3p	1.525028935
hsa-miRPlus-E1038	1.515845807
rno-miR-19b	1.452562327
rno-miR-133a	1.439674083
rno-miR-26b	1.434479419
rno-miR-384-3p	1.431055901
rno-miR-129*	1.413793103
rno-miR-20b-5p	1.410658307
rno-miR-181a	1.402365501
hsa-miR-1264	1.400736335
rno-miR-34a	1.392950761
hsa-miRPlus-F1222	1.390770252
rno-miR-100	1.389264877
rno-miR-23a	1.388569875
hsa-miR-1297	1.385174419
rno-miR-20a	1.378527947
rno-miR-17-5p	1.372731855
rno-let-7b	1.370829659
rno-miR-423	1.362669246
rno-miR-148b-3p	1.338410596
mmu-miR-1274a	1.311891746
rno-miR-186	1.298256538
hsa-miR-1274b	1.287296898
hsa-miR-1280	1.282561405
rno-miR-29c	1.450121246
rno-miR-24	1.301975641
rno-miR-181d	1.289013075
rno-miR-290	1.257022123

**Table 4:** Networks of molecules identified from IPA analysis of the 30 miRNA list.

ID	Molecules in Network	Score	Focus Molecules
1	EIF2C2,hydrogen peroxide,INS,mir-17,mir-19,mir-24,mir-29,mir-30,mir-34,mir-99,mir-133,mir-154,mir-181,mir-186,mir-219,mir-290,miR-148b-3p/miR-148a-3p/miR-152 (includes others),miR-17-5p/miR-20b-5p/miR-93-5p (includes others),miR-181a-5p/miR-181b-5p/miR-181a (includes others),miR-186-5p/miR-186,miR-18b-5p/miR-18a-5p/miR-18a (includes others),miR-19b-3p/miR-19b/miR-19a-3p,miR-219-2-3p,miR-219-5p,miR-24-3p/miR-24,miR-29a-5p/miR-29a*,miR-382-5p/miR-382,miR-409-5p,miR-487b/miR-487b-3p,miR-494/miR-494-3p,miR-539-5p/miR-539,miR-99a-5p/miR-100-5p/miR-99b-5p (includes others),MYLIP,PPARA,STAT3	47	18
2	25-hydroxy-vitamin D3,ARID4B,BNIP2,CCNE2,CCNF,DBF4 (includes EG:10926),DGCR8,DROSHA,EPHB6,estrogen receptor,FSH,Gulo,hCG,let-7,let-7a-3p/let-7f-1-3p/let-7b-3p (includes others),MAP2K1/2,MICA,mir-15,mir-17,mir-23,mir-26,mir-30,mir-181,mir-1297,miR-17-5p/miR-20b-5p/miR-93-5p (includes others),miR-181a-5p/miR-181b-5p/miR-181a (includes others),miR-18b-5p/miR-18a-5p/miR-18a (includes others),miR-20a-3p/miR-20a*,miR-26a-5p/miR-26b-5p,MYLIP,PHF6,PNPT1,TRIB1,Vegf,XPO5	25	11

### Bioinformatics analysis of microRNA downstream effector genes

Using miRWALK, of the >20,000 non-unique targets, we identified 56 unique targets in our differentially expressed gene list (N = 256) which paired, through sequence complementarity, with 8 miRNAs. Bicluster analysis with the 56 genes and 8 miRNAs [19] predicted networks of miRNA and mRNA module involving growth hormone [20], Hepatocyte growth factor (HGF), Invariant polypeptide of major histocompatibility complex (CD74) that is enriched in biological function involving positive regulation of peptidyl-tyrosine phosporylation (GO:0050731), P = 1.43e-0.05.

The IPA miRNA target filter identified 5 miRNAs targeting 3 genes from our 70 gene list. MiR-148-3p, mir-17-5p, miR-181a-5p, miR-19b-3p and miR-24-3p were predicted to control the expression of the following target genes: Interleukin 6 signal transducer IL6ST (gp130). I kappa b kinase epsilon (IKBKE) and DnaJ (Hsp40) homolog, subfamily C, member 21 (DNAJC21). Specific predicted interactions are listed in Table 5.

**Table 5:** List of predicted interactions between miRNA from the 39 list and genes from the 70 genes list, using the Ingenuity miRNA target filter.

Symbol	Fold Change	Confidence	Symbol
miR-148b-3p (and other miRNAs w/seed CAGUGCA)	1.338	High (predicted)	IL6ST
miR-17-5p (and other miRNAs w/seed AAAGUGC)	1.411	High (predicted)	IL6ST
miR-181a-5p (and other miRNAs w/seed ACAUUCA)	1.402	High (predicted)	DNAJC21
miR-19b-3p (and other miRNAs w/seed GUGCAAA)	1.453	High (predicted)	IL6ST
miR-24-3p (and other miRNAs w/seed GGCUCAG)	1.302	Moderate (predicted)	IKBKE

In addition, we used DNA Intelligent Analysis (DIANA) tools to further analyze targets for the miR17-5p. Mapk14 and Stat3 were identified as targets with prediction scores of 0.727 and 0.901 and verified by 5 and 4 methods, respectively. Next, we performed an analysis of expression data for microRNA function using the DIANA0mirExTra tool. Il6ST (gp130) was identified by the DIANA-microT target prediction tool as a gene target for mir17-5p.

### Molecular validation of the miRNA predicted gene targets

**Fig 4:**
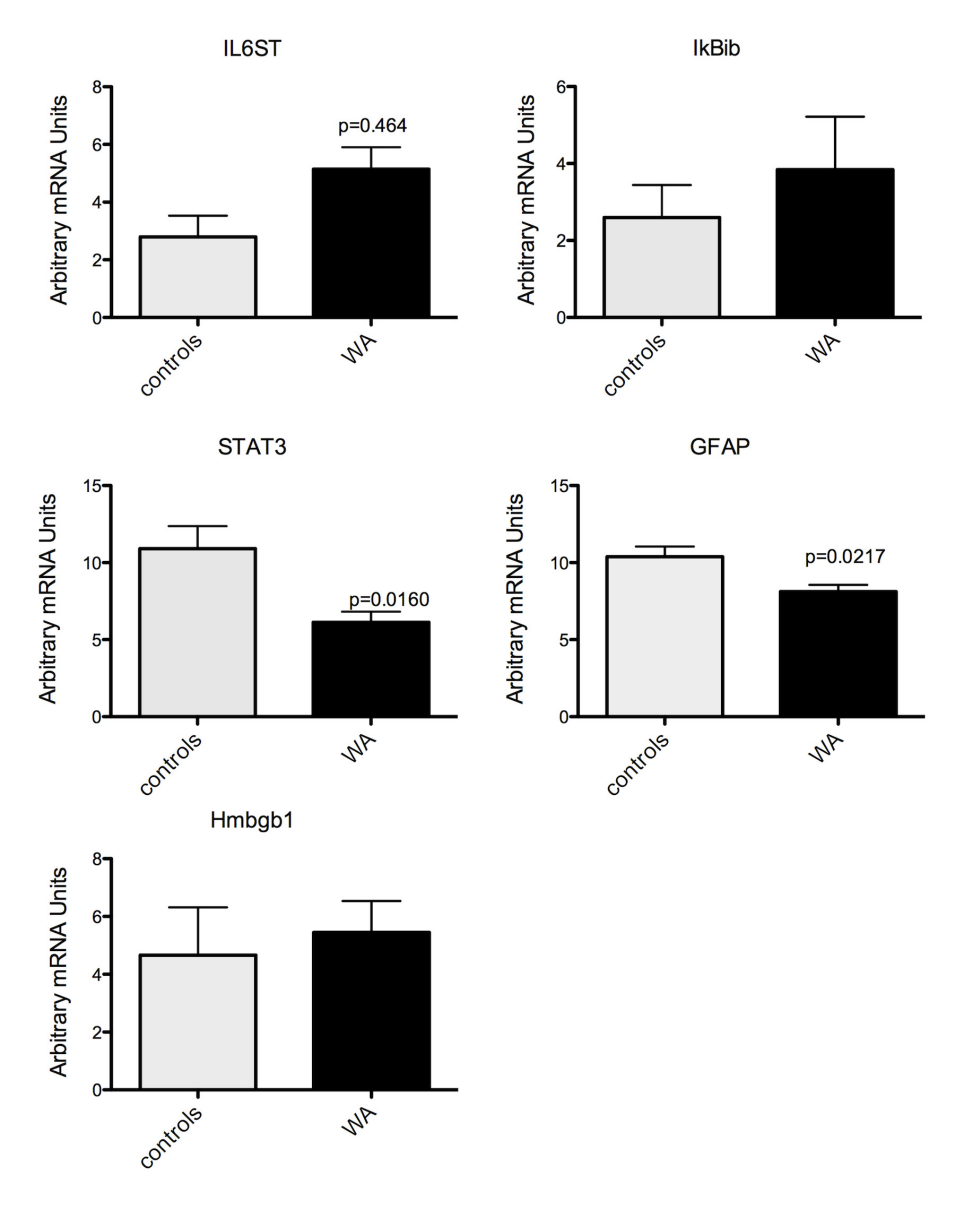
Real Time RT-PCR verification of selected gene expression after chronic WA stress. **IL6ST was significantly up-regulated in stress samples compared to controls while both STAT3 and GFAP were significantly reduced.** P<0.5, n = 4 in each group.

**Fig 5:**
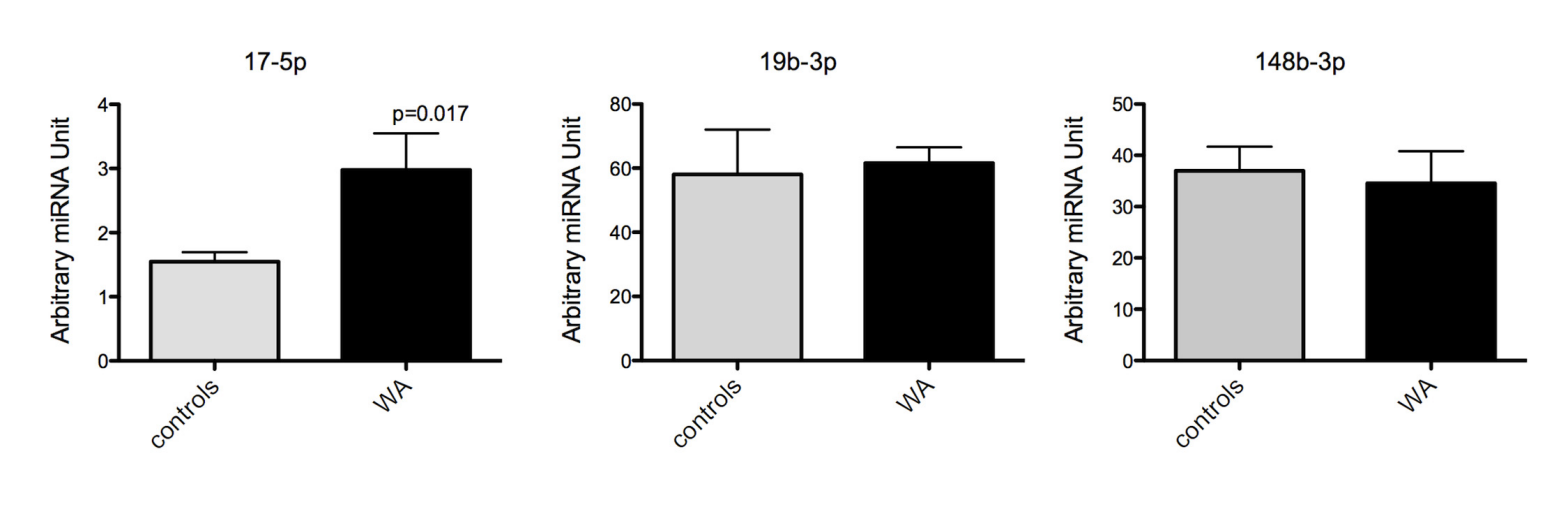
Real Time RT-PCR verification of selected miRNA expression after chronic WA stress. Mir17-5p was significantly up-regulated in samples from stressed rats compared to controls. P<0.5, n = 4 in each group.

To confirm changes in mRNA and miRNA expression level observed from our arrays, q-RT-PCR was performed in duplicates from the same RNA samples used for microarray and additional samples from the same experiment. Q-RT-PCR was performed on selected genes and miRNA that were identified in networks and pathways of interest described above. We measured the expression of the Interleukin 6 signal transducer gp130 (IL-6ST), I kappa b kinase epsilon (IKBKE), GFAP, high mobility group box 1 (Hmbg1), signal transducer and activator of transcription 3 (acute-phase response factor (STAT3) and mir17-5p, 148-3p and 19b-3p. We observed a significant increase in IL6ST (gp130) in samples from stressed rats compared to controls (Fig 4). STAT3 and GFAP expression were significantly decreased in stressed rats compared to controls (Fig 4). While there was a trend for an increased expression of IKBKE and Hmbg1 in stressed rats compared to control, the changes were not significant (Fig 4). We also found a significant increase in the expression of mir17-5p in stressed rats compared to controls. In contrast, there was no change in the expression of mir148-3p or mir19b-3p (Fig 5).

### In mir17-5p situ hybridization with co-immunofluorecent staining for GFAP

**Fig 6:**
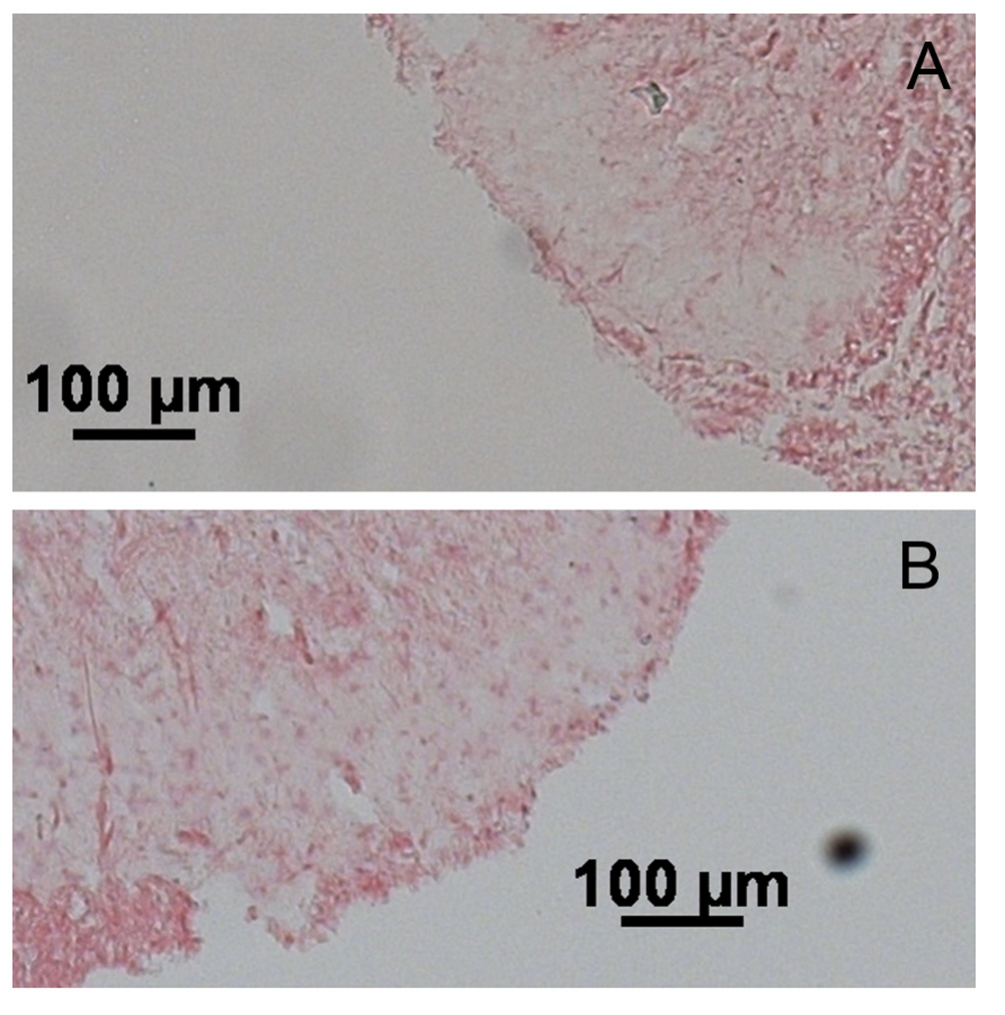
Representative images of mir-17-5p In situ hybridization in the dorsal horn of the spinal cord using the NBT-BCP detection system. A) Spinal cord isolated from control rats. B) Spinal cord isolated from stressed rats. Increased mir-17-5p staining is observed in the dorsal horn. Scale bar is 100mM.

**Fig 7:**
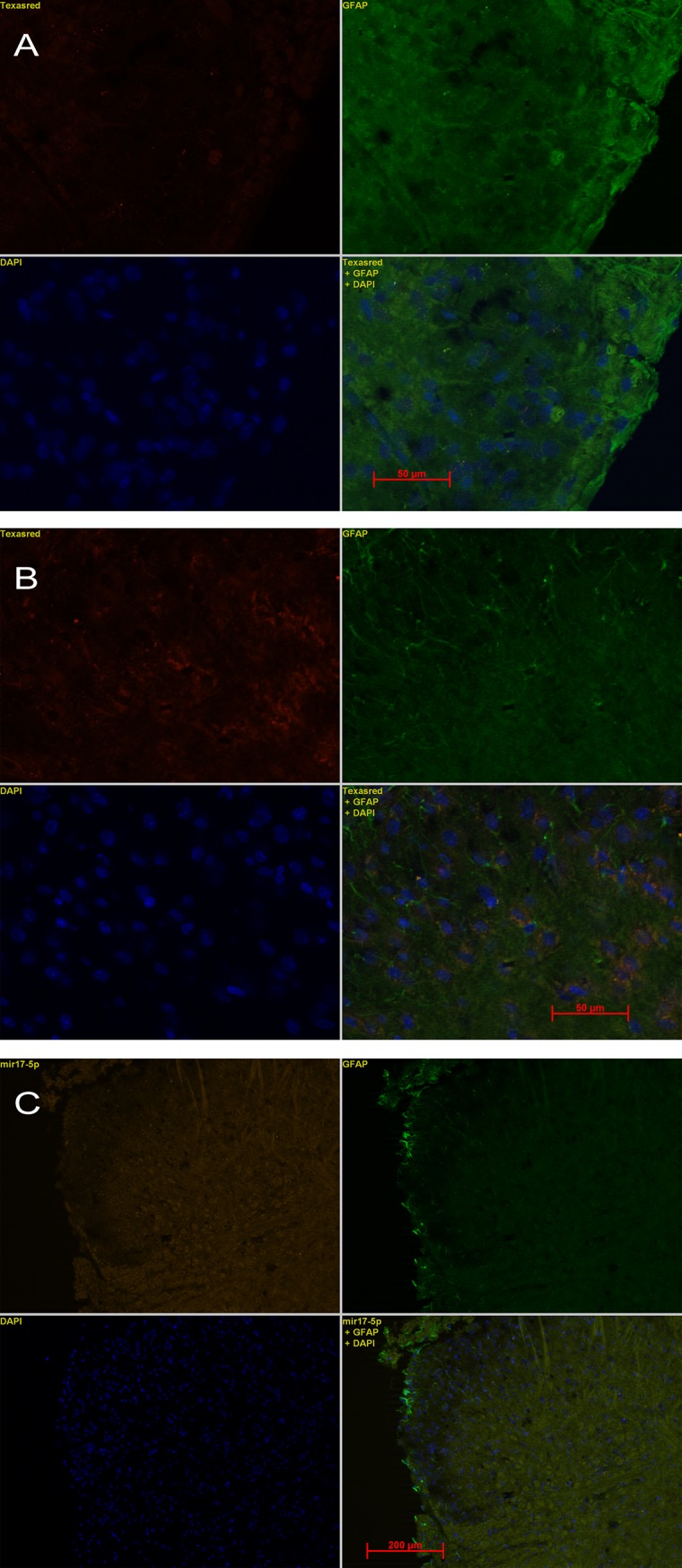
Expression of mi17-5p in the spinal cord assessed by in situ hybridization with co-immunofluorescence staining for astrocytes (GFAP) and DAPI. A) In situ hybridization of mir17-5p (red) with co-immunostaining for GFAP (green) and DAPI (blue) in the dorsal horn of the spinal cord from control rats. B) In situ hybridization of mir17-5p (red) with co-immunostaining for GFAP (green) and DAPI (blue) in the dorsal horn of the spinal cord from stressed rats. Arrows indicate evidence of mir-17-5p staining in peri-nuclear space colocalizing with astrocytes. Scale car = 50mM. C) In situ hybridization with scramble negative control probe with co-immunofluorescence staining for astrocytes (GFAP) in the dorsal horn of the spinal cord from a stressed rat. Scale bar = 200 mM.

The expression and localization of mir17-5p in the spinal cord was evaluated using in situ hybridization for mir17-5p. Sections stained with the NBT-BCP detection system showed high mir17-5p expression in the dorsal horn of the spinal cord from stressed rats (Fig 6). When using the HNPP Fluorescent detection set for fluorescent detection of mir17-5p combined with immunofluorecent labeling for GFAP for astrocytes, staining for mir17-5p was verified in sections from stressed animals and while the expression was observed throughout the spinal cord, mir17-5p was also clearly expressed in the superficial laminae of the dorsal horn spinal cord where nociceptive fibers from the gut are distributed. The co-immunostaining with GFAP revealed co-localization of mir17-5p with astrocytes, but also in the peri-nuclear region of other cells as shown by mir17-5p staining in DAPI positive cells (Fig 7A and 7B). As expected, sections treated with the scramble negative control probe showed no specific staining (Fig 7C)

### Effect of the miRCURY LNA mir17-5p inhibitor on stress-induced visceral hyperalgesia

**Fig 8:**
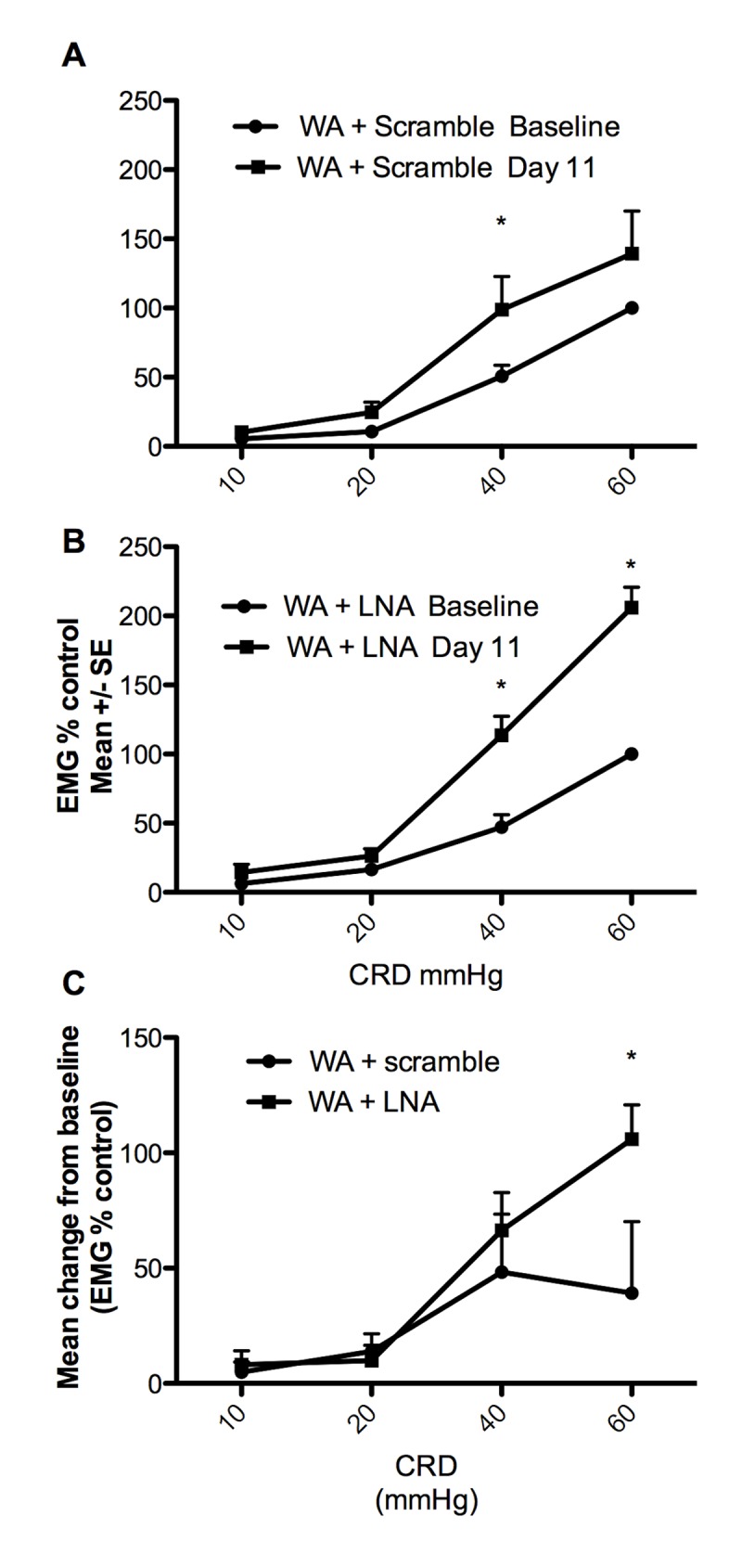
Effect of the miRCURY LNA mir17-5p inhibitor or scramble control on stress-induced visceral hyperalgesia. A) EMG response to graded CRD at baseline and 24 hours following the 10^th^ stress session showed overall increase of the EMG response to CRD with significant increased at 40 mmHg consistent with stress-induced visceral hyperalgesia n = 8 *P<0.05 two-way ANOVA followed by Bonferroni post test. B) Stressed rats treated with the LNAmir17-5p inhibitor exhibit increased EMG response to CRD at day 11 compared with baseline indicating visceral hyperalgesia n = 6, *P<0.05 two-way ANOVA followed by Bonferroni post test. C) The mean change from baseline was significantly higher in stressed rats treated with the LNAmir17-5p inhibitor (n = 6) compared with stressed rats treated with the control scramble (n = 8) indicating an exacerbation of visceral sensitivity *P<0.05 two-way ANOVA followed by Bonferroni post test.

Stressed rats treated with the scramble negative control probe showed significant increase of VMR to CRD at day 11 compared with baseline at 60 mmHg. These results are consistent with our previous report that chronic WA induces visceral hyperalgesia[13]. Interestingly, rats exposed to WA stress and receiving the treatment with the miRCURY LNA mir17-5p inhibitor probe exhibited exacerbated increase of the VMR to CRD compared with the scramble control (Fig 8).

## Discussion

The current study demonstrates that chronic water avoidance stress in rats, which we have previously characterized as a model of visceral hypersensitivity and anxiety[14], induces changes in the expression of a network of spinal genes and miRNAs involved in neuroinflammation. Our findings, suggests an effect of stress on a signaling pathway involving mir17-5p upregulation and changes in gene expression of IL6ST and STAT3 (known to affect the astrocytic GFAP gene transcription). These results support our previous findings showing that this stress model is characterized by strong phenotypic changes in spinal glia, in particular astrocytes, which we previously found play an important role in the sensitization of the visceral nociceptive pathways[15].

### Chronic WA stress causes changes in mRNA expression in the lumbar spinal cord

In the present study, gene network and gene ontology analyses, in the genes differentially expressed after stress, revealed two networks related to inflammatory diseases as well as canonical pathways related to IL-6, PI3K/AKT and acute phase response signaling. IL6ST was a candidate gene of interest overlapping in all 3 pathways. These results showing activation of networks with a strong immune component after stress, are consistent with our previous results showing changes in key pro-inflammatory mediators in the circulation and in the spinal cord after chronic water avoidance stress such as an increase in the level of cytokines IL-6 and IL-1ß in the spinal cord early in the course of the 10 days WA stress[15]. Similar observations of increased IL-6 expression in the CNS and at the periphery in response to stress have been reported in both rodents and humans [21,22].

IL6ST, also known as gp130, is an essential component of signal transduction for the family of IL-6-type cytokines including IL-6, IL-11, IL-27, Leukemia inhibitory factor (LIF), oncostatin M (OSM), ciliary neurotrophic factor (CNTF), B cell stimulating factor (BSF)3 and cardiotrophin (CT)1[23]. Upon ligand binding, gp130 dimerization induces intracellular signaling cascades that involve Janus kinases (JAKS), Signal Transducer and Activator of Transcription (STATs) and the tyrosine phosphatase SHP2/Akt, all involved in the regulation of various cellular processes such as proliferation, differentiation and gene activation[24]. STAT1/3 dimers formed after phosphorylation by JAKS are known to translocate to the nucleus where they can modulate the transcriptional activation of more that a 1000 target genes as shown in cultured astrocytes[25]. In a recent study, an important role of the JAKS and STAT3 signaling pathways has been demonstrated in the proliferation of astrocytes in the dorsal horn of the spinal cord in a model of peripheral nerve injury in rats [9]. Though, the exact mechanisms underlying this effect remain unknown, STAT3-mediated regulation of the transcriptional activation of astrocytic genes encoding for cell cycle proteins has been proposed to play a significant role. Interestingly, STAT3 associated with other transcription factors may be involved in the transcriptional activation of GFAP in astrocytes [9]. In our model, we were able to verify using qRT-PCR, a significant increased in IL-6ST expression (gp130) in spinal samples from stressed rats compared to controls while STAT3 and GFAP expression was significantly decreased in stressed rats compared to controls. These results suggest a possible down-regulation of the STAT3 pathways leading to decreased transcription of GFAP. While these results are in line with our previous findings showing a decrease of spinal GFAP protein expression after stress, the inverse relationship between IL6ST and STAT3 observed in terms of gene expression is surprising as a positive activation of JAK/STAT3 signaling has been described in other studies [26]. However, It is possible that the changes observed are the results of a combination of factors such as changes in JAKS activity and STAT3 phosphorylation leading a negative modulation of downstream genes such as GFAP. Changes in SOCS3 (Suppressor of Cytokines Signaling) may also account for a negative regulation of the JAK/STAT3 pathway despite increased expression of IL6ST. SOCS3 is part of a family of STAT-induced STAT inhibitors (SSI), which are cytokine-inducible negative regulators of cytokine signaling, which can act as an anti-inflammatory signal [20] or in our study as an inhibitor of STAT3-induced regulation of GFAP expression. This hypothesis has not been tested in our model. In addition to its role in the regulation of inflammation, gp130 has been involved in the transduction signals of pathological pain as in experimental arthritis [27]. An essential role of gp130 in sensory neurons in the maintenance of experimentally-induced mechanical hypersensitivity has been recently demonstrated [28]. The precise implication of the CNS gp130/STAT3 pathways in visceral nociception signaling has not been investigated.

### Chronic WA stress causes change in micro-RNA expression in the lumbar spinal cord

Emerging interest in the role of microRNA in mediating stress responses has prompted a new field of investigation looking at the impact of stress on the biogenesis of miRNAs, the expression of their mRNA targets and the activities of miRNA-protein complexes [reviewed in [5]]. In our model, we identified 39 miRNAs affected by stress, as part of 2 main networks both including inflammatory diseases as top functions. Based on the knowledge that miRNAs and their target genes are connected in networks, we performed an analysis of the miRNA predicted target genes looking for miRNA and target genes in overlapping networks in our miRNA and gene lists. Amongst the 5 miRNAs (miR148-3p, miR17-5p, miR181a-5p, miR19b-3p and miR24-3p) targeting multiple genes from our 70 genes list, mir17-5p, which is increased with stress was confirmed by qRT-PCR. Our initial bicluster analysis confirmed the 17-5p as a key player and highlights the potential role of this specific miRNA in glial modulation in the spinal cord in our stress model. In situ hybridization confirmed high expression of mir17-5p with stress in the dorsal horn of the spinal cord and the partial co-localization of mir17-5p with GFAP (labeling astrocytes) supports a potential modulatory role of mir17-5p on glial activity and neuro-immunomodulation. CD74 is a membrane protein that acts as a chaperone for major histocompatibility complex (MHC) class 2 molecules but also as a receptor-binding site for the macrophage migration inhibitory factor (MIF) which bears great influence on the CNS cellular and molecular inflammatory status[29]. mir17-5p was of particular interest as its mRNA targets identified via the Ingenuity miRNA target filter, include IL6ST and STAT3. However, while the general knowledge on miRNA suggest a down-regulation of target genes, we observed an increase in IL6ST and a decrease in STAT3, both measured by qRT-PCR. Several possible mechanisms may be at play and account for these results. For example, miRNA-mediated repression of gene expression depends on multiple factors such as the relative concentration of miRNAs and mRNA targets or the number of mRNAs targeted by the same miRNA. The repressive activity of miRNA can also be affected by RNA-binding proteins or miRNA-protein complexes binding to mRNA. [30]. It is difficult to assess the mechanisms involved in IL6ST up-regulation in our study. However, our analysis highlights the effect of stress on the IL6ST/STAT3 signaling pathway in the spinal cord, which has been previously involved in pain and inflammatory diseases as well as spinal astrocytes activity. The expression of mir17-5p in the dorsal horn of the spinal cord where convergence of nociceptive signals from the gut occurs, supports our hypothesis that stress-induced alteration in the network connecting mir17-5p and the IL6ST/STAT3 system may be involved in the modulation of visceral sensitivity. This was confirmed by our results showing that treatment with a miRCURY LNA mir17-5p inhibitor during stress was able to modulate visceral hypersensitivity compared with a control scramble treatment. However, although the exacerbation of the visceral sensitivity after treatment with the mir17-5p inhibitor was unexpected, it does not invalidate our hypothesis but illustrates the complexity of the system as miRNAs may affect the expression of multiple targets with opposing effects on nociceptive signaling which will be evaluated in further profiling studies.

While our study provides novel insight on the modulatory effect of chronic stress on a network of genes and miRNA involved in inflammatory pathways related to IL6/gp130/STAT3/GFAP, there are limitations. Our samples were whole spinal cord segments, and while we showed mir17-5p expression co-localization with astrocytes in the dorsal horn of the spinal cord in stressed animals, we were unable to assess cellular specificity for our complete gene/miRNA expression data set. The specific role of astrocytes in the effect of stress on the JAK/STAT3 pathways (as suggested by prior studies showing JAK/STAT3 regulating spinal astrocytes proliferation and neuropathic pain in rats) is of great interest and further studies requiring transgenic animals are necessary to perform similar gene and miRNA arrays in isolated neuronal or glial cells from the spinal cord of adult stressed rats. In future studies, transfection of neuronal or glial cells with specific miRNA shown to change with stress may help characterizing specific mirRNA-gene signaling circuitry potentially affected by stress at the cellular level. Finally, it is important to consider that while several publications have documented the effect of stress in rodents on genes and miRNAs expression in various tissues[31], there are no comparative studies assessing genomic and epigenetic changes across various stress models. The knowledge that specific signaling systems are consistently affected by stress would provide an important basis to better define the pathophysiological consequences of stress and characterization of therapeutic targets.

**Fig 9:**
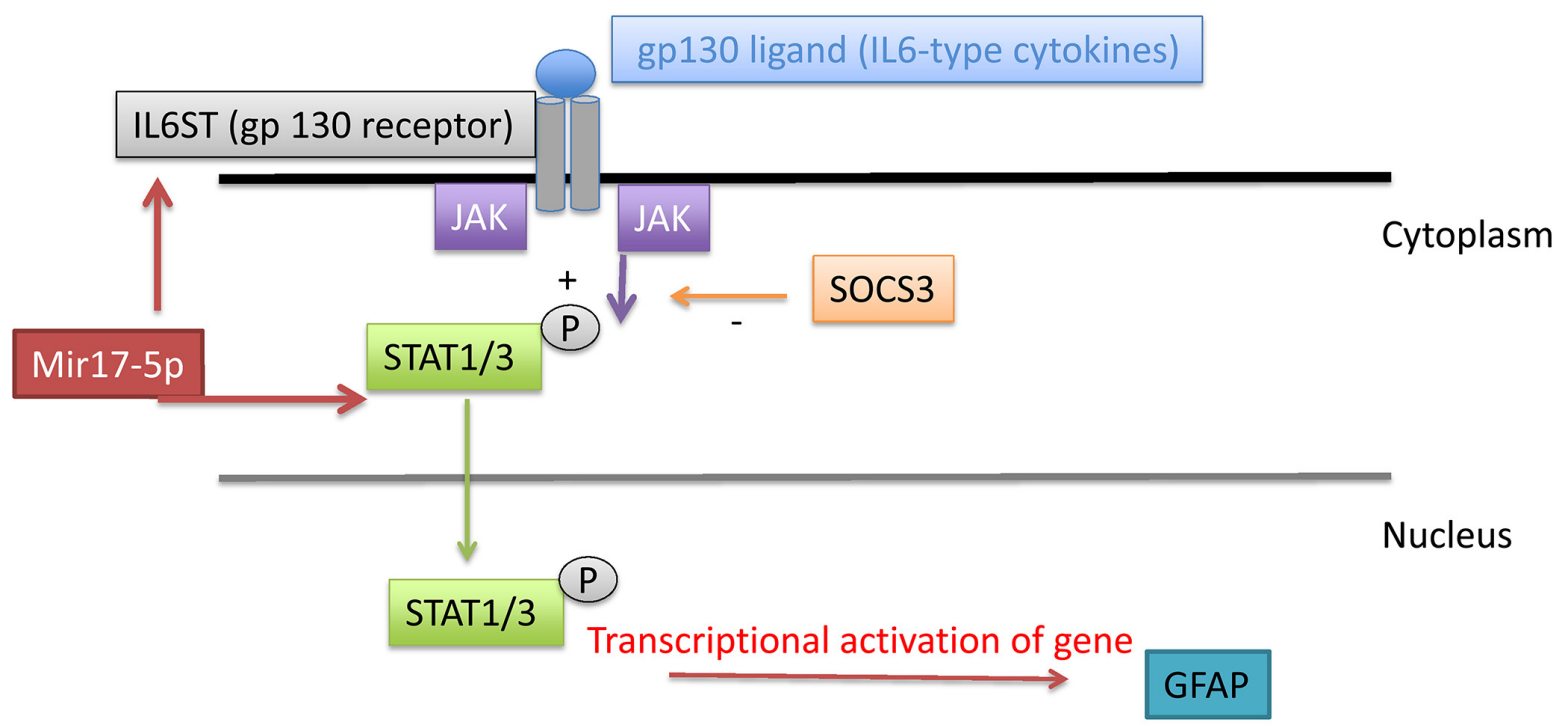
Schematic representation of the modulation of the IL6ST/STAT3/GFAP system in our model. Binding of IL-6-type cytokines to gp130 induces gp130 dimerization, and subsequent intracellular signaling cascades leading to STAT1/3 phosphorylation by JAKS. After dimerization and translocation to the nucleus, STAT1/3 act as a transcriptional factor for multiple genes including GFAP. SOCS3 may act as an inhibitor of STAT3-induced regulation of GFAP via inhibition of STAT1/3 phosphorylation. Mir17-5p is a potential regulator of the system by modulating the expression of IL6ST and STAT3.

In conclusion, we demonstrated, using a high throughput method, that chronic stress affects several gene-miRNA networks involved in inflammatory pathways relevant to pain signaling in the spinal. Our findings, suggesting a link between mir17-5p upregulation and changes in gene expression of IL6ST and STAT3 (known to affect GFAP gene transcription, as summarized in Fig 9), provide new insight into the possible mechanisms mediating the effect of chronic stress on neuro-inflammation in the spinal cord.
